# The effects of exercise training on lipid profile in patients with sarcoidosis

**DOI:** 10.1038/s41598-021-84815-4

**Published:** 2021-03-10

**Authors:** Dariusz Jastrzebski, Beata Toczylowska, Elzbieta Zieminska, Aleksandra Zebrowska, Sabina Kostorz-Nosal, Elzbieta Swietochowska, Camillo Di Giulio, Dariusz Ziora

**Affiliations:** 1 Department of Lung Diseases and Tuberculosis, Faculty of Medical Sciences in Zabrze, Medical University of Silesia, 41-800 Zabrze, Poland; 2 Nalecz Institute of Biocybernetics and Biomedical Engineering, Polish Academy of Sciences, 02-109 Warsaw, Poland; 3 Mossakowski Medical Research Centre, Polish Academy of Sciences, 02-106 Warsaw, Poland; 4 Institute of Sport Sciences, Department of Physiological and Medical Sciences, Academy of Physical Education, 40-065 Katowice, Poland; 5 Department of Medical and Molecular Biology, Faculty of Medical Sciences in Zabrze, Medical University of Silesia, 41-808 Zabrze, Poland; 6 Department of Neuroscience and Imaging, University of Chieti-Pescara, Via dei Vestini 31, 66100 Chieti, Italy

**Keywords:** Physiology, Biomarkers, Diseases, Health care, Medical research

## Abstract

This study aimed to determine the use of lipid profiling to assess the effects of moderate intensity exercise training (ET) on patients with sarcoidosis. Fourteen patients with sarcoidosis (mean age, 46.0 ± 9.6 years) were examined before and after 3-week of ET programme in hospital settings. Symptoms (fatigue: FAS, dyspnoea: MRC), lung function tests and physical function tests (6 MWT, muscle force) were measured before and after ET. Proton nuclear magnetic resonance (NMR) spectroscopy combined with orthogonal partial least squares-discriminant analysis (OPLS-DA) was used to determine lipid profile before and after ET. Twenty-five NMR signals from lipid compounds were selected for further analysis as well as serum lipid and inflammatory markers. Three weeks of ET results in improvement of symptoms (FAS: 27.5 vs. 21.0; p < 0.001, MRC: 0.86 vs. 0.14; p = 0.002) and physical function (6MWT: 508.43 vs. 547.29; p = 0.039). OPLS-DA analysis of the lipid profiles of patients with sarcoidosis revealed differences among the samples before and after ET, including decreases in fatty acids (p < 0.017), triglycerides (p < 0.022) and total cholesterol (p < 0.020). Other changes included shifts in fatty acids oxidation products and triacylglycerol esters. A short-time, in-hospital exercise training benefits patients with sarcoidosis by enhancing their physical function. Additionally, positive effect on lipid profile was observed also in this study. It is suggested that lipid profiling could become a new prognostic method to assess effects of pulmonary rehabilitation in patients with sarcoidosis.

## Introduction

Sarcoidosis is a multisystem inflammatory disease in which the immune system cells differentiate and proliferate contributing to granuloma formation in several tissues^1–3^. The disease is characterized by a hyperimmune response in which excessive secretion of inflammatory mediators and uncontrolled oxidative stress have a detrimental effect on the mitochondria function and amino acid metabolism in myocytes and on lipid profiles^4–6^. These alterations in myocytes lead to myopathy and contribute to the development of fatigue, a common complain among sarcoidosis patients^4^. The interaction of all these factors is dynamic and complex and is not dependent on a single factor. The symptoms of sarcoidosis are generally nonspecific and include general weakness, arthralgia, reduced exercise capacity, dyspnea, and fatigue. Changes in serum and lipoprotein lipid levels and their metabolism as a consequence of inflammation may indicate an increased risk for atherosclerosis^7,8^. Hence, chronically elevated inflammatory-immune response is a major risk factor for neuromuscular and cardiovascular dysfunction^1,2,8^. Thus, maintaining serum and lipoprotein lipid levels near the recommended range and regular physical activity (PA) are recommended as an integral part of sarcoidosis treatment and prevention of hyperlipidemia-related complications^9^.

An increasing number of studies investigated the effect of exercise-induced alterations of the human metabolite responses in physiological and pathological conditions^10,11^. Small to large-fold changes were reported for metabolites related to oxidative stress, glycolytic pathways, and fatty acid metabolism, leading to a better understanding of the biological impact of physical activity, such as post-exercise variations in plasma fatty acids, ketone bodies, fatty acid oxidation products, sulfated bile acids, triacylglycerol esters, bile acids and minor phospholipids (lysophosphatidylcholines and lysophosphatidylethanolamines). Metabolic data may improve scientific understanding of the complex beneficial effect that physical activity has on the related molecular mechanisms in patients with multivariate aetiology of fatigue symptom, such as in patients with granulomatous diseases, as sarcoidosis^12^.

Previous studies focusing on the lipid profile status in pulmonary sarcoidosis demonstrated that the pathogenesis of sarcoidosis has been associated with alterations in the lipid profile including the reduction in high-density lipoprotein cholesterol levels, lower apolipoprotein A1 levels and higher oxidative stress^8,12^. Exercise intolerance, which is defined as an inability or decreased ability to perform physical exercise is influenced in pulmonary sarcoidosis by multiple factors, including sarcoidosis-related skeletal muscle abnormalities, decreased pulmonary function, small fibre neuropathy, and increased risk of atherosclerosis^13–15^. Reduced physical activity is consistently associated with poorer outcomes, including higher mortality risk^8,15–17^.

It has been previously reported that the quality of life may be partially amenable to physical exercise training as part of a comprehensive pulmonary rehabilitation program^18,19^. Supervised physical training programs improve exercise performance and fatigue among patients with sarcoidosis^18^. Improvements in skeletal muscle function and oxidative capacity lead to reduced ventilatory requirement for a given submaximal load and decreased dyspnea. Exercise training may have other positive effects, including changes in lipid metabolism, increased antioxidant protection and reduced inflammation^3,6,19^.

Relatively few clinical trials have evaluated the metabolite changes in response to exercise training program in lung diseases^10,11^. A few studies have identified markers involved in lipid metabolism that determine the risk of progression to pulmonary diseases^20–22^. Telenga et al.^21^ reported higher expression of lipids from the sphingolipid pathway in smokers with chronic obstructive pulmonary diseases (COPD) compared to smokers without COPD. In the study of Yan et al.^22^, analysis of lipid profile from 22 patients with idiopathic pulmonary fibrosis and 18 control subjects revealed that the glycerophospholipids (GPs) are the important biological molecules for the backbone of cellular membranes. Landi et al.^23^ reported an important analytical role of proteomics approach in identifying lipid components to the comprehension of sarcoidosis. In the study of Toczylowska et al.^24^ it has been showed that levels of several proteins involved in lipid metabolism (e.g.: phosphatidylcholine, triglycerides, fatty acids and sphingomyelin) are expressed differently in sarcoidosis patients than in controls.

Considering these lipid metabolite modifications observed in patients with sarcoidosis it seems interesting to establish whether lipid profile analysis can be used to verify the potential health benefits of physical exercise training. Therefore, this study aimed to investigate whether moderate intensity training benefits patients with sarcoidosis and to determine the usefulness of lipid profiling in assessing the effects of exercise training program.

## Methods

### Study design and subjects

In this observational study, the outcomes of sarcoidosis were compared among patients who completed an exercise training program. All patients provided informed consent for the study, which was conducted in accordance with the guidelines of the 2008 revision of the Declaration of Helsinki and the Research Ethics Committee of the Medical University of Silesia, Poland (KNW/0022/KB1/123/15). The research was registered in the Australian New Zealand Clinical Trials Registry (ACTRN12619001479190).

**Scheme 1:**
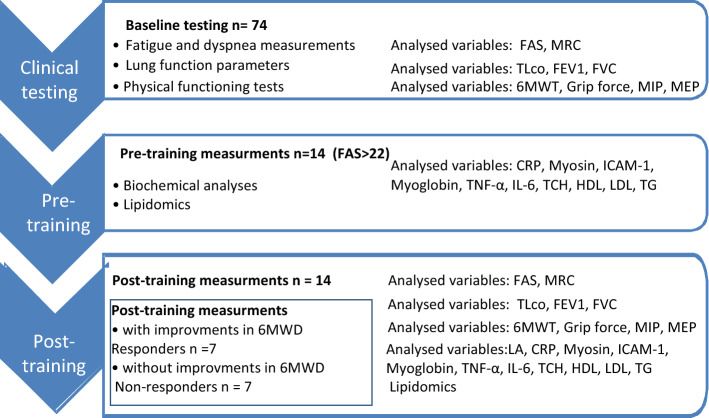
The manner of analysing the selected variables.

Out of the 74 patients with sarcoidosis treated at the Department of Lung Diseases and Tuberculosis between January 2014 and December 2017, fourteen patients who fulfilled the following criteria were selected for this study: with confirmed pulmonary sarcoidosis (ATS/ERS criteria)^3^, no evidence of comorbidities, no evidence of extra pulmonary sarcoidosis, no evidence of history of smoking, and no current use of corticosteroids or other immunosuppressive agents. Moreover, only patients with a history of fatigue (Fatigue Assessment Scale > 22) with newly diagnosed (up to 6 months) sarcoidosis were included in this study (Table 1). There were about 22.0 percent of patients with BMI above the normal range (> 24.9 kg/m^2^). The entire list of patients BMI and total cholesterol can be found as Supplementary Table S1 online. The baseline serum metabolic variables didn’t show significant differences in patients with normal BMI and in overweight patients. The patients were diagnosed based on consistent clinical features, bronchoalveolar lavage (BAL) fluid analysis, and/or biopsy-proven non-caseating epithelioid cell granulomas^3^. For the entire duration of the experiment, all patients were asked to consume the same low-fat balanced diet in hospital settings. No caffeine, antioxidants supplements, and alcohol were permitted 48 h before and during the experiment. The experiment design has been presented in the Scheme 1.

**Table 1:** Somatic characteristics of the patients with sarcoidosis.

Variables	Sarcoidosis group n = 14
Age (years)	46.0 ± 9.6
Height (m)	1.7 ± 0.4
Weight (kg)	78.6 ± 8.8
BMI (kg/m^2^)	27.2 ± 4.7 kg/m^2^

*BMI* Body Mass Index.

### Fatigue and dyspnea measurements

Fatigue was measured using the 10-item Fatigue Assessment Scale (FAS). Each item uses a five-point rating scale with a total score range of 10–50. Scores below 22 indicate no fatigue, scores of 22–34 indicate mild–moderate fatigue, and scores of 35 or more indicate extreme fatigue. The minimal clinically important difference (MCID) in sarcoidosis is four points or a 10% change^25^.

The Medical Research Council (MRC) questionnaire was used to determine the level of dyspnea. For descriptive and statistical reasons, the MRC questionnaire was modified so that the shortness of breath, except for strenuous exercise, was marked as grade 1^26^.

### Lung function parameters

Lung function tests were performed using a lung test apparatus and included an assessment of forced vital capacity (FVC), forced expiratory volume during the first second (FEV_1_, lung test 1000) and transfer factor for carbon monoxide (TL_CO_, MedGraphic Plethysmograph).

The pulmonary function tests were conducted in accordance with the current European Respiratory Society (ERS) recommendations^27,28^.

### Assessment of physical functioning

To estimate patients’ physical function, the 6-min walk test (6MWT) was used. The test was performed in accordance with the 2015 Polish Respiratory Society guidelines^29^. On the basis of American Thoracic Society/European Respiratory Society (ATS/ERS) technical standards, 33 m was adopted as the MCID^30^. In order to identify the patients’ susceptibility to the training stimulus, the delta differences between 6MWT distance after intervention and before intervention were calculated (Δ). Taking 6MWT MCID (minimal clinically important difference) into consideration, the patients were classified after rehabilitation into responders (n = 7, Δ6MWT > 33 m) and non-responders (n = 7, Δ6MWT < 33 m).

The measurement of maximal isometric grip strength (1 RM test) was conducted in accordance with the recommendations of the American Society of Exercise Physiology (ASEP)^31^. The maximal isometric grip force of both hands was measured with a Meden-Inmed Baseline Hydraulic Hand Dynamometer (Meden-Inmed, Poland). Each participant performed three maximum voluntary contractions (1 RM tests) for each hand. The test always started with the dominant hand. A timed rest break of 30 s was given between each trial and each hand received 1 min rest break before proceeding to the next trial. Patients were instructed to stop squeezing when they felt pain or discomfort during measurement. The participants were encouraged to perform to invest maximal effort during the tests. The average of three tests was calculated and used in training intensity analysis. Muscle force of left and right hand before and after exercise training intervention were compared.

Respiratory muscle force was measured by estimating the maximal inspiratory pressure (MIP) and maximal expiratory pressure (MEP) in accordance with the ATS/ERS guidelines^31^, using the Threshold IMP (Healthdyne Technologies, Great Britain). The functional assessment tests were used to plan the intensity of physical training program in patients with sarcoidosis.

### Physical training protocol

Patients were encouraged to start a 3-week moderate intensity exercise training program (5 days a week), which was supervised by a physical therapist and based on their physical performance assessed at baseline. In accordance with the ATS standards, the exercise program comprised three components:

1. aerobic endurance training (stationary cycling or treadmill up to 30 min/day) with intensity of 70–80% of HRmax calculated in accordance with Karvonen formula {(target heart rate = [(maxHR – restingHR × %intensity] + [restingHR)},
2. inspiratory muscle training, using the Threshold IMP (Healthdyne Technologies, Great Britain) and consisted of six courses of inspiratory exercises with five inspiratory maneuvers in each series and 1 min of rest between them. The level of inspiratory load was calculated based on the initially measured MIP values, which is in accordance with the ATS/ERS guidelines^32^; likewise, MEP values were used to calculate the inspiratory load. The patients were required to start the exercises at 30% of the maximal levels measured during the initial MIP and MEP measurements.
3. peripheral muscle strength training (three sets of 15–20 repetitions of five different exercises with intensity about 30% 1RM)^33^. The resistance level was individualized for each patient (in accordance with patient performance), reassessed, and adjusted after every session using the Borg Rating of Perceived Exertion. Pulse oximetry was used to monitor peripheral oxygen saturation levels during exercise, and supplemental oxygen use during training was commensurate with current prescriptions when required.

In summary, the patients performed a short, 3-week, moderately intensive (2 h/day), in-hospital rehabilitation program consisting of endurance exercise, respiratory muscle training and peripheral muscle strength training. Physiological and biochemical variables were measured before and after completing the exercise training.

### Biochemical analysis

Blood samples were collected after overnight fasting at baseline and after the 3-week exercise training. Blood samples were collected in evacuated tubes containing EDTA (ethylenediaminetetraacetic acid) and serum sample tubes after a 12 h fasting period. Serum was separated via centrifugation at 1500*g* for 10 min at 4 °C. Aliquots of each sample were stored at − 80 °C. The samples were thawed before analysis. Myosin and myoglobin were determined by immunoenzymatic assays. Serum myosin levels were measured with Bio-Vendor LLC test (BioVendor, Czech Republic) and serum myoglobin (MB) levels using Human Myoglobin Enzyme Immunoassay (Myoglobin, ELISA kit, GMBH, Germany) and the clinical chemistry analyzer Universal Microplate Spectrophotometer-µQUANT (Bio-Tek World Headquarters, USA). The intra-assay coefficients of variation (CV) for these assays were 2.8% and 4.6%, respectively.

C-reactive protein (CRP) was assessed in serum by a turbidimetric immunoassay using a Dade-Behring (Deerfield, IL, USA) kit (intra assay CV 3.45%). Total cholesterol (TCH), triglycerides (TG), low-density lipoprotein concentration (LDL-c), and high-density lipoprotein concentration (HDL-c) were assayed using enzymatic methods and the clinical chemistry analyzer (RA-XT, Technicon Instruments Corporation, USA). The intra-assay CV for these assays were below 5.0%. Atherogenic index of plasma (AIP) was calculated as mathematical relationship between TG and HDL (log TG/HDL-C) and used as a significant predictor of atherosclerosis. In addition, the markers of inflammation and cell damage (ICAM-1, intercellular adhesion molecule-1; TNF-α, tumor necrosis factor α; IL-6, interleukin-6) were analyzed in the collected samples. Serum ICAM-1 levels were assessed by the immunoenzymatic method with Quantikine Immunoassay (R&D Systems, USA). Serum TNF- α and IL-6 were measured by the immunoenzymatic method using tests DTA00D and D6050 Quantikine Immunoassay (R&D Systems, USA). The intra-assay CV for these assays were 3.06%, 3.0% and 2.1%, respectively.

### Lipid profile study design

Lipidomics is a subset of metabolomics used for the measurement of specific metabolites—the hydrophobic compounds^10,11^. In the current study, proton NMR spectroscopy of the hydrophobic part of serum was performed to obtain a metabolic profile. Data from the analyzed spectra were used for lipid profiling using univariate and multivariate statistical analyzes.

Lipids were extracted from 500 μL of serum samples (frozen and stored at − 80 °C and thawed before extraction) using the modified Bligh and Dyer method^34^, by adding 36% HCl for extraction mixture in proportion 0.05:1:2 v/v HCl:methanol:chloroform. The chloroform phase of the sample was dried using nitrogen. Dry residues were then diluted in 600 μL of CDCl3 and immediately tested^35^. All NMR spectra were acquired at 20 °C using a Varian Inova 400 (Varian Inc., USA) spectrometer. The proton NMR spectra were collected using a standard one-pulse sequence (5 s delay time, 90° pulse, and 128 repetitions). Zero filling to 32 k data points, line broadening of 0.5, and baseline and phase correction were applied to each spectrum using the existing software in the spectrometer.

### Data analysis

Routine biochemical compounds were analyzed using matched-pairs *t* test or Wilcoxon matched-pairs signed-rank test (univariate) and orthogonal partial least squares-DA (OPLS-DA) and OPLS-effect projection (OPLS-EP; multivariate tests)^36^.

Quantities of metabolites were expressed in terms of relative intensity (based on the magnitude of the spectral peak and relative to the chloroform signal) using home written software. The measured signal magnitudes reflect to the concentrations of the compounds. We selected 25 signals of the NMR spectrum for the statistical analysis of the lipid extracts.

Signal assignments were made based upon our own database and data from the literature^37–40^. Spectra of compound have one or several signals but they all change in the same direction with compound concentration changes. Therefore, we selected the most isolated signals belonging to the particular compound or functional chemical group. Other signals from the same compounds, mostly overlapped with other signals, were ignored. Exception were complex signals: from plasmalogen and triglycerides (PL/TG), from phosphatidylcholine (PC) and sphingomyelin (SM) (PC/SM), from saturated fatty acid (FA), monounsaturated FA and polyunsaturated FA and PC, SM, PE (PE- phosphatidylethanolamine).

Mean centering, log transformation, and Pareto scaling were applied before the discriminant analysis (OPLS-DA). In addition, responses to the exercise training as changes of variables before and after training were assessed. The OPLS-EP test was separately carried out for the routine biochemical analysis and NMR lipid data.

### Multivariate projection method for data exploration

OPLS was used to interpret the systematic changes existing among samples characterized by the relative concentrations of many of the metabolites. In the OPLS-DA, the goodness of fit is reported as the cumulative score across all of the components of R2cum—explained by the model and Q2cum—as predicted by the model. The OPLS-DA model was considered significant if R2cum and Q2cum were significantly larger than zero and good when both values were equal or greater than 0.5^41–43^.

The variable importance in projection (VIP) value of each variable in both models was calculated to indicate its contribution to the classification. Variables with VIP values greater than 1.0 were considered significantly different, and larger VIP values represented higher contributions to discrimination between groups. The model was validated by applying the analysis of variance testing of cross-validated predictive residuals (CV-ANOVA) test.

Multivariate analysis (OPLS-DA) was performed using the SIMCA-P software package (Version 12, Umetrics AB, Sweden)^42^.

### Ethics approval and consent to participate

This study was conducted in accordance of the Declaration of Helsinki and approved by the Research Ethics Committee of the Medical University of Silesia, Poland (KNW/0022/KB1/123/15). The research was registered in the Australian New Zealand Clinical Trials Registry (ACTRN12619001479190). Written informed consent were obtained from all participants.

## Results

Statistical analysis showed significant changes in clinical features after 3-week physical training program (Table 2). After completing the rehabilitation program, patients with sarcoidosis reported lower fatigue using FAS scale (p < 0.001) and MRC scores (p = 0.002). Significant effect was also found for TL_CO_ (%pred) (p = 0.026) and 6-min walking distance (p = 0.039). Significant positive effect of exercise training was observed for the maximal isometric grip strengths and the respiratory muscle force (Table 2). Univariate statistical analysis of changes in biochemical parameters before and after exercise training showed significant differences in myosin (p = 0.003), ICAM-1 (p = 0.015), TNF-α (p < 0.001), IL-6 (p < 0,001), TCH (p = 0.031), HDL (p = 0.006), and TG (p < 0.001) (Table 3). The value of atherogenic index of plasma (AIP) showed high cardiovascular risk levels (0.37)^44^. Exercise training significantly reduced AIP values (p < 0.008) despite the apparent nonsignificant changes in the serum LDL or CRP levels. Moderate Spearman’s Rank correlations were noted between the responders to rehabilitation and Δ6MWT and MRC (*r* = − 0.63), also and 6MWT (*r* = 0.69), and HDL-c (*r* = 0.83).

**Table 2:** Clinical features of the patients with sarcoidosis at baseline and after exercise training.

Variables	Baseline assessment	After intervention	p-value
**Fatigue and dyspnea measure**			
FAS	27.50 ± 5.28	21.00 ± 4.41	<** 0.001**
MRC	0.86 ± 0.66	0.14 ± 0.36	**0.002**
Lung function tests			
TL_CO_ (% pred.)	74.43 ± 16.94	76.14 ± 15.39	**0.026**
FEV_1_ (%pred.)	95.14 ± 10.20	93.00 ± 12.13	0.808
FVC (% pred.)	98.43 ± 14.75	99.57 ± 14.41	0.503
FEV_1_%/FVC	97.43 ± 9.65	97.86 ± 6.41	0.217
**Physical function tests**			
6MWT (m)	508.43 ± 79.89	547.29 ± 70.62	**0.039**
Grip force RH (kg)	34.60 ± 12.90	37.90 ± 12.60	**0.004**
Grip force LH (kg)	32.20 ± 13.20	35.70 ± 12.90	<** 0.001**
MIP (mmH_2_O)	59.86 ± 15.69	74.50 ± 15.74	**0.014**
MEP (mmH_2_O)	67.21 ± 23.92	90.71 ± 19.72	**0.002**

Data are expressed as absolute number or mean ± standard deviation (SD).

*FAS* Fatigue Assessment Scale, *MRC* Medical Research Council questionnaire, *TL*_*CO*_ transfer factor for carbon monoxide, % of predicted, *FEV*_*1*_ forced expiratory volume during the first second, *FVC* forced vital capacity, *6MWT* six-minute walk test, *RH* right hand, *LH* left hand, *MIP* maximal inspiratory pressure, *MEP* maximal expiratory pressure.

**Table 3:** Biochemical data.

Variables	Baseline assessment	After intervention	p-value
CRP (mg/L)	3.00 ± 4.62	2.45 ± 2.40	0.358
Myosin (µg/mL)	1.73 ± 0.27	5.61 ± 1.97	**0.003**
ICAM-1 (ng/mL)	468.25 ± 42.10	462.87 ± 48.29	**0.015**
Myoglobin (ng/mL)	75.15 ± 10.68	91.71 ± 8.31	0.063
TNF-α (pg/mL)	10.37 ± 2.18	8.71 ± 2.13	<** 0.001**
IL-6 (pg/mL)	12.67 ± 0.74	9.43 ± 0.88	<** 0.001**
TCH (mmol/L)	6.97 ± 0.58	6.60 ± 0.52	**0.031**
HDL (mmol/L)	1.27 ± 7.07	1.35 ± 0.65	**0.006**
LDL (mmol/L)	4.34 ± 0.44	4.27 ± 0.46	0.108
TG (mmol/L)	1.89 ± 0.22	1.56 ± 0.19	<** 0.001**
AIP	0.37 ± 0.23	0.32 ± 0.09	**0.008**

*CRP* C-reactive protein, *ICAM-1* intercellular adhesion molecule-1, *TNF-α* tumor necrosis factor alpha, *IL-6* interleukin 6, *TCH* total cholesterol, *HDL* high-density lipoprotein, *LDL* low-density lipoprotein, *TG* triglyceride, *AIP* atherogenic index of plasma (AIP).

**Figure 1:**
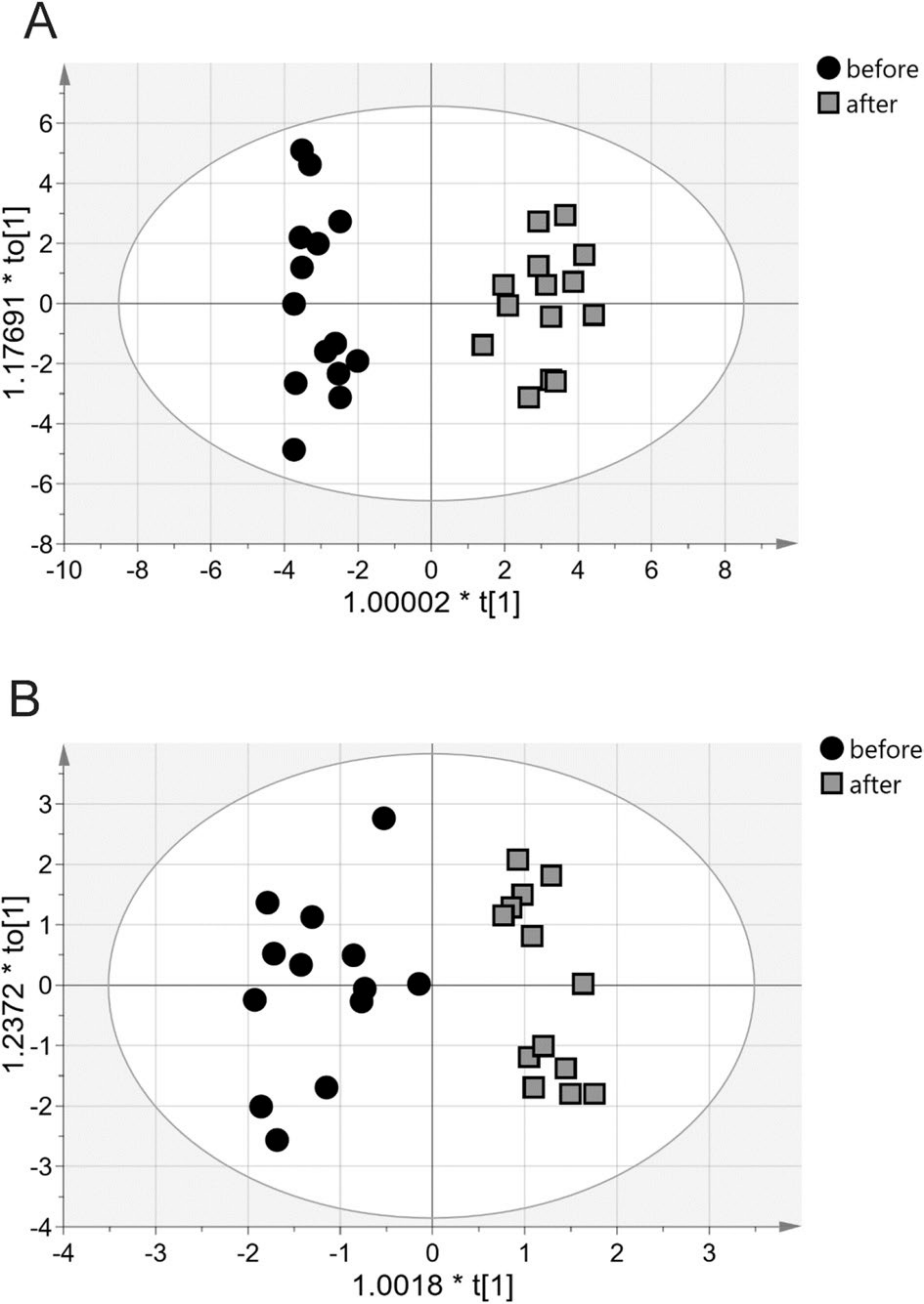
Score plots of the two-component OPLS-DA. (**A**) Biochemical clinical data of before and after exercise training groups. (**B**) NMR spectroscopy lipids data of before and after exercise training groups. t_o_[1] represents variations within class for the first orthogonal component, whereas t[1] represents variations between classes for the first predictive component. Ellipse represents Hotelling's T2 with 95% confidence interval. *OPLS-DA* orthogonal partial least squares discriminant analysis, *NMR* nuclear magnetic resonance.

Likewise, OPLS-DA analysis of routine biochemical compounds (CRP, HDL, LDL, TNF-α, IL-6, myosin, myoglobin, TG, TCH, and ICAM) showed differences between the patients before and after exercise training (Fig. 1A). Mean centering and Pareto scaling were applied before model building. The OPLS-DA model consisted of one predictive and one orthogonal component, and the R2X and Q2cum values were 0.73 was 0.79, respectively. The CV-ANOVA test yielded a p-value of 0.016. The misclassification table indicated that all patients (100%) were accurately classified into their groups (Fishers exact probability, 0.0011). The most important parameters that influenced the differentiation of the groups (VIP value > 1) were myosin and IL-6. After exercise training, myosin levels increased, whereas IL-6 levels decreased.

OPLS-DA analysis of the lipid profiles of patients with sarcoidosis revealed differences among the samples before and after exercise training. The built model consisted of one perpendicular and two orthogonal components with R2Xcum and Q2cum values of 0.80 and 0.81, respectively, indicating good data fit and prediction. According to the misclassification table, 96.43% of the subjects were appropriately classified into groups (Fishers exact probability, 3.7e−7; 100% before and 92.86% after rehabilitation; Fig. 1B). The CV-ANOVA test yielded a p-value of 5.96e−006.

In the lipid profile analysis, the most important parameters for group differentiation (VIP > 1) were the following: complex signal PL/TG (5.27 ppm), complex signal phosphatidylcholine/sphingomyelin (PC/SM; 3.65 ppm), fatty acid (FA) (5.38, 1.84, 1.62, 1.30, 1.26, and 0.89 ppm), TCH (1.50, 1.13 ppm), free cholesterol (FC; 1.0 ppm), and complex signal of PC/SM/PE (4.05 ppm) (Table 4).

**Table 4:** Selected compound/functional groups in the NMR spectra and the percent changes in their NMR signal intensities.

Chemical shift (ppm)	Compound/functional group	After vs. before (%)	ANOVA p-value	VIP value > 1
5.56	SM	70.1	0.241	–
5.38	FA	44.4	**0.017**	1.00
5.27	PL/TG	38.2	<** 0.001**	1.37
4.64	EC	66.1	**0.020**	–
4.36	PC	38.1	**0.030**	–
4.29	TG	62.9	**0.022**	–
4.05	PC/SM/PE	63.4	0.262	1.39
3.65	PC/SM	21.9	**0.003**	1.51
3.50	Pregnenolone	121.8	0.496	–
3.38	PC	117.3	0.459	–
2.81	PUFA	92.9	0.240	–
2.76	PUFA	55.0	**0.027**	–
2.32	Acyl groups in FA	56.31	0.322	–
2.04	FA (palmitic acid)	77.6	0.191	–
1.84	Acyl groups in FA	52.5	**0.011**	1.03
1.62	Acyl groups in FA	57.3	**0.017**	1.14
1.50	TCH	52.9	**0.020**	1.05
1.30	Saturated FA, MUFA and PUFA	51.9	**0.017**	1.08
1.26	Saturated FA	63.7	**0.035**	1.01
1.13	25-Hydroxycholesterol	51.3	<** 0.001**	1.12
1.03	EC	81.0	0.296	–
1.0	FC	56.9	**0.011**	1.02
0.89	Unsaturated ω-6 acyl groups and FA	34.9	<** 0.001**	1.26
0.86	FC	84.0	0.173	–
0.71	TCH	101.98	0.852	–

*SM* sphingomyelin, *FA* fatty acids, *PL* plasmalogen, *TG* triglycerides, *EC* cholesterol esters, *PC* phosphatidylocholine, *PE* phosphatidylethanolamine, *PUFA* polyunsaturated fatty acids, *TCH* total cholesterol, *FC* free cholesterol.

In addition, the response to the exercise training of the patients with sarcoidosis and the parameters that showed significant alterations after exercise training were analyzed using the OPLS-EP method^35^. This method was most suitable for comparing dependent samples. The model for analysis of routine clinical data consisted of one predictive component, and the R2Xcum and Q2cum values were 1.0 and 0.99, respectively, demonstrating excellent data fit and prediction. Among the routinely measured biochemical compounds, sICAM-1 intercellular adhesion molecule-1 and myosin were most altered after rehabilitation due to changes in their higher absolute values.

**Figure 2:**
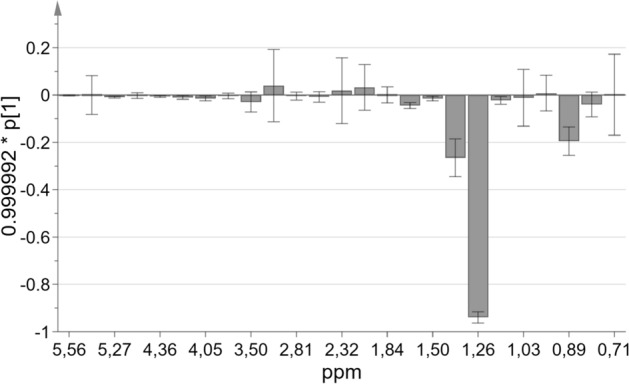
OPLS-EP loading plot of lipids profile changes after rehabilitation (NMR spectroscopy data in ppm of compound signal chemical shift); p[1] corresponds to the covariance between the NMR data and the predictive score vectors. *OPLS-EP* orthogonal partial least squares effect projection, *NMR* nuclear magnetic resonance.

Lipid profile data obtained from the NMR spectra and analyzed using OPLS-EP allows to obtain a model consisting of one predictive and three orthogonal components (R2cum, 0.93; Q2cum, 0.86; p = 0.01). VIP results revealed that the levels of FA and cholesterols (total and esterified) and SM changed after rehabilitation in the patients with sarcoidosis (Fig. 2). This figure is the loading plot of variables in OPLS-EP analysis. We can indicate variables direction of their changes after rehabilitation.

**Figure 3:**
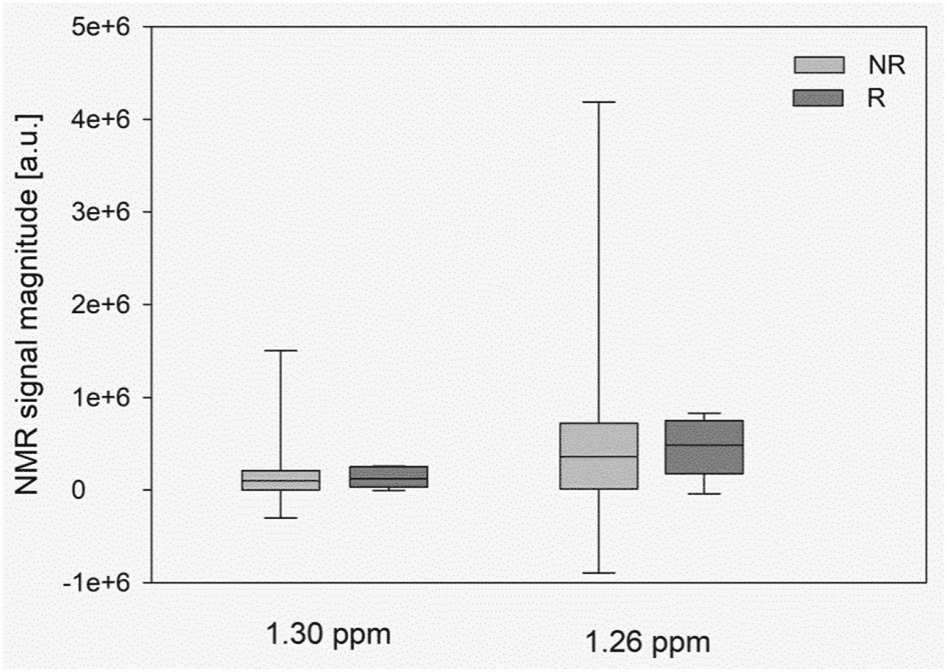
Mean ± SD signal magnitude changes of the most changed after exercise training fatty acids NMR at 1.26 ppm and 1.30 ppm. *NR* non-responders, *R* responder patients, *FA* fatty acids, *SD* standard deviation, *NMR* nuclear magnetic resonance.

Figure 3 shows the mean ± standard deviation (SD) NMR signal magnitude changes of the two signals of FAs, which were the only with the VIP value > 1 in the OPLS-EP analysis. They magnitudes of these signals increased compared with those of the other compound signals.

## Discussion

To the best of our knowledge, this is the first study to compare the lipid profiles of patients with sarcoidosis before and after an exercise training program using systemic and lipidome-wide analysis on serum lipids.

A major finding of the study is that moderate exercise training significantly reduced proinflammatory cytokines, increased secretion of the factors regulating muscular function and modified serum lipid profile. Other important findings include, statistically significant beneficial effects of exercise program on patient’s physical functioning and greater resistance to fatigue. All aforementioned exercise-induced changes were associated with higher expression of proteins involved in lipid metabolism.

Sarcoidosis is a multisystem inflammatory disease, which can affect all organs, nevertheless most patients present respiratory symptoms, such as dyspnea, fatigue and exercise limitation^1,4,5^. A possible cause of fatigue in sarcoidosis patients is activation of their autoimmune genetic background and multiple inflammatory-immune pathways that exert a systemic inflammation. Other causes for a “reversible” fatigue may be metabolic disorders, and psychosocial conditions, such as depression, anxiety, and stress^1,6,15,16^.

Indeed, in the present study patients with sarcoidosis reported lower fatigue using FAS scale and MRC scores after 3 weeks of exercise training compared to the baseline levels. The increased exercise tolerance, higher HDL levels and reduced AIP values and cytokines (TNF-α and IL-6) in response to exercise training suggested a positive effect of the rehabilitation program on cardiovascular risk in sarcoidosis patients. Since the evaluation of proinflammatory cytokines is a valuable tool for evaluation of the risk of developing disease-related complications^1,8,45,46^, the study findings suggest that the after exercise training sarcoidosis patients may be at the lower risk of developing such complications.

Identification of the lipid metabolite levels by lipid profile allowed better understanding of metabolic reactions in response to physical training in these patients^10,12^. Therefore, it is thought that the changes in the complex signal PL/TG, complex signal phosphatidylcholine/sphingomyelin (PC/SM), fatty acid (FA) (5.38, 1.84, 1.62, 1.30, 1.26, and 0.89 ppm), TCH (1.50, 1.13 ppm), free cholesterol (FC), and PC/SM/PE (4.05 ppm) may contribute to lipids effects of exercise in sarcoidosis patients.

Lipidomics is the large-scale study of the acquisition of lipid compositions in biological systems, where lipids are broadly defined as hydrophobic or amphipathic small molecules that may entirely or partially originate from carbanion-based condensation of thioesters and/or from carbocation-based condensations of isoprene units^45^. The role of lipids in respiratory disease has attracted more attention in recent research data including IPF, COPD and sarcoidosis, the diseases which are associated with metabolic complications^46–48^.

In our previous systematic and lipids analysis of profiles of serum lipids in patients with sarcoidosis^24^, the VIP analysis showed that the elements that were most important for differentiation between patients with sarcoidosis and healthy individuals were PC/SM, TCH, TG, FC, SM, FA, and pregnenolone. FC (0.86 ppm) and PC/SM (3.65 ppm) had high statistical reliabilities as discriminating variables (putative biomarkers). Wide spectrum of the individual lipid molecules also has the ability to differentiate idiopathic pulmonary fibrosis (IPF) from controls^46^. The levels of screened glycerophospholipids (GPs), which are important biological molecules for the backbone of cellular membranes, decreased in patients with IPF when compared with the control subjects. Among the subcategories of GPs, two cardiolipins (CLs), two glycerophosphatidic acids (Pas), 13PC, two glycerophosphoethanolamines (PEs), one glycerophosphoinositol (PI), and four glycerophosphoserines (PSs) were identified as unique lipids in patients with IPF based on the VIP scores. Moreover, three out of the 46 kinds of sterol lipids and one out of the 20 types of FAs were considered potential biomarkers^22^.

As already stated in this study, sarcoidosis is often accompanied by alterations in lipoprotein profiles^19^ and an increased risk of atherosclerosis^8^. Decreased levels of HDL cholesterol (HDL-c) and apolipoprotein A1 in active disease without any significant changes in TCH, LDL cholesterol (LDL-c), and TG have been observed in untreated sarcoidosis^8,46^. In the study by Vekic et al., patients with sarcoidosis had fewer LDL I subclasses (p = 0.001) but increased LDL II and III subclasses (p = 0.001) when compared with the controls^47^. This pattern was evident in acute and chronic disease groups. The patients also had smaller HDL size (p = 0.001) and higher proportions of HDL 2a (p = 0.006) and 3a particles (p = 0.004). Furthermore, patients with chronic sarcoidosis had smaller LDL size when compared with those with acute disease (p = 0.02) and higher proportions of HDL 3a subclasses (p = 0.04) than the controls.

In acute sarcoidosis, the relative proportions of LDL and HDL particles were associated with the levels of inflammatory markers, whereas in chronic disease, an association with the concentrations of serum lipid parameters was found. The obtained results demonstrated an adverse lipoprotein subfraction profile in sarcoidosis with sustained alterations during the disease course. Thus, the evaluation of LDL and HDL particles may be helpful in identifying patients with a high cardiovascular risk at least for prolonged corticosteroid therapy due to chronic diseases^19^. Moreover, the improvement of metabolites related to the tricarboxylic acid cycle and bioenergetics pathway following moderate intensity exercise may be an important indicator of the assessment of the rehabilitation and treatment of sarcoidosis patients.

In a proteomic study, BAL (bronchoalveolar lavage) proteins involved in the regulation of lipid metabolism (such as apolipoprotein A1 (apoA1) and serum amyloid A) were found to be differently expressed in patients with sarcoidosis when compared with the controls^23^. The proteins downregulated in sarcoidosis included plastin-2 and fatty acid-binding protein 4. Lipid profile analysis can identify serum amyloid A in BAL fluid and serum, demonstrating that this acute-phase protein is overexpressed in sarcoidosis^48^.

The results of the present study indicate for the first time that the lipid profiles of patients with sarcoidosis can change during a 3-week exercise training program. Only a few studies have investigated exercise-induced lipid profile changes following regular physical training^10^. In patients with coronary heart disease, aerobic exercise for 8 weeks led to a significant decrease in serum concentrations of TG and apoC3 (apolipoprotein C3) when compared with baseline levels^49^. In a recent meta-analysis of 25 healthy Asians, the weighted mean differences in HDL-C, TCH, and TG estimated in serum significantly improved after regular aerobic exercises^46^. However, Fikenzer et al. stated that unselected training intervention studies showed only minor positive effects on HDL-C and TG and did not exert significant effects on serum LDL-C^50^. Only effective endurance training for a duration of 40–50 min per training unit 3–4 days/week over a period of at least 26 weeks showed improvements in serum lipid levels. These studies employed different training protocols and study designs, and performed metabolomics in subjects with different health status. Therefore, future lipid profile analysis based exercise training study will improve scientific understanding of the quantitative effect of physical activity on serum lipids.

The diagnostic importance of serum lipid profile was presented in our previous study^24^, where the lipid profile of patients with sarcoidosis was established and compared to that of physically active healthy subjects. The study found that lipid profiles of healthy individuals were different from those of patients with sarcoidosis. Our present study revealed positive effects of rehabilitation program consisting of endurance, peripheral muscle strength, and respiratory muscle training on exercise tolerance. which may play a role in the prevention of sarcoidosis complications. The effectiveness of the rehabilitation program of sarcoidosis patients was confirmed by higher myosin levels, lower proinflammatory cytokine levels and the beneficial changes in lipid profiles estimated by lipidomics. We observed significant lower atherogenic index of plasma (AIP) in patients after rehabilitation. It has been suggested that AIP values of − 0.3 to 0.1 are associated with low, 0.1 to 0.24 with medium and above 0.24 with high risk for atherosclerosis^44^. Exercise training significantly reduced AIP values (p < 0.008) despite the apparent non-significant changes in the serum LDL or CRP levels. Moreover, AIP differences in response to exercise training have been of great value in assessment of beneficial changes in patients’ cardiovascular risk^44^. This observation suggests that the addition of endurance and resistance exercise to rehabilitation program of individuals with sarcoidosis may help to reduce the risk of developing complications and mortality. Finally, the current study revealed some metabolite differences in response to exercise training and a few promising markers which may play a role in monitoring effectiveness of the pulmonary rehabilitation in patients with sarcoidosis.

There are several limitations in this research study. Firstly, it has a small sample size and the lipid profile response to exercise is not compared with healthy subjects. Secondly, it is not a longitudinal study, which made it impossible to observe the clinical impacts of the intervention on the lipid profile. Moreover, we do not have information about the diet that patients had prior to the study. Thus, further longitudinal multicenter studies using larger samples are required to establish whether evaluation of lipid profile biomarkers can be used as diagnostic and prognostic tools.

## Conclusions

Our results suggest that moderate intensity exercise training has beneficial effects on patients’ exercise tolerance, serum proinflammatory cytokine levels, and lipid profile which might be important determinants for the prevention of sarcoidosis complications. Analysis of exercise-induced alterations in many lipids of sarcoidosis patients may become a new prognostic method to assess effectiveness of pulmonary rehabilitation, but more investigations are needed to establish that.

## Supplementary Information

Supplementary Table S1.

## Data Availability

All datasets are available from the corresponding author on reasonable request.

## Abbreviations

6MWT: Six-minute walk test
apoC3: Apolipoprotein C3
BAL: Bronchoalveolar lavage
CRP: C-reactive protein
EC: Cholesterol esters
ERS: European Respiratory Society
FA: Fatty acids
FAS: Fatigue Assessment Scale
FC: Free cholesterol
FEV_1_: Forced expiratory volume during the first second
FVC: Forced vital capacity
HDL: High-density lipoprotein
ICAM-1: Intercellular adhesion molecule-1
IL: Interleukin
LDL: Low-density lipoprotein
LH: Left hand
MCID: Minimal clinically important difference
MEP: Maximal expiratory pressure
MIP: Maximal inspiratory pressure
MRC: Medical Research Council
NMR: Nuclear magnetic resonance
OPLS-DA: Orthogonal partial least squares-discriminant analysis
PE: Phosphatidylethanolamine
PC: Posphatidylocholine
PL: Plasmalogen
PUFA: Polyunsaturated fatty acids
RH: Right hand
SM: Sphingomyelin
TCH: Total cholesterol
TG: Triglycerides
TL_CO_: Transfer factor for carbon monoxide
TNF: Tumor necrosis factor

## References

[1] Grunewald JA (2019). Sarcoidosis. Nat. Rev. Dis. Prim..

[2] Iriate A, Rubio-Rivas M, Villalba N, Corbella X, Mañá J (2020). Clinical features and outcomes of asymptomatic pulmonary sarcoidosis. A comparative cohort study. Respir. Med..

[3] Costabel U, Hunninghake GW (1999). ATS/ERS/WASOG statement on sarcoidosis. Sarcoidosis Statement Committee. American Thoracic Society. European Respiratory Society. World Association for Sarcoidosis and Other Granulomatous Disorders. Eur. Respir. J..

[4] Drent M, Lower E, De Vries J (2012). Sarcoidosis-associated fatigue. Eur. Respir. J..

[5] Cho PSP (2019). Physical inactivity in pulmonary sarcoidosis. Lung.

[6] Ivaniševic J (2012). Dyslipidemia and oxidative stress in sarcoidosis patients. Clin. Biochem..

[7] Simonen P, Lehtonen J, Gylling H, Kupari M (2016). Cholesterol metabolism in cardiac sarcoidosis. Atherosclerosis.

[8] Bargagli E (2017). Increased risk of atherosclerosis in patients with sarcoidosis. Pathobiology.

[9] Wallert B (2020). Long-term effects of pulmonary rehabilitation on daily life physical activity of patients with stage IV sarcoidosis: A randomized controlled trial. Respir. Med Res..

[10] Sakaguchi CA, Nieman DC, Signini EF, Abreu RM, Catai AM (2019). Metabolomics-based studies assessing exercise-induced alterations of the human metabolome: A systemic review. Metabolites..

[11] Han X (2016). Lipidomics for studying metabolism. Nat. Rev. Endocrinol..

[12] Mirsaeidi M (2016). Plasma metabolomic profile in fibrosing pulmonary sarcoidosis. Sarcoidosis Vasc. Diffuse Lung Dis..

[13] Marcellis RG, Lenssen AF, de Vries J, Drent M (2013). Reduced muscle strength, exercise intolerance and disabling symptoms in sarcoidosis. Curr. Opin. Pulm. Med..

[14] Marcellis RGJ, Lenssen AF, Kleynen S, De Vries J, Drent M (2013). Exercise capacity, muscle strength and fatigue in sarcoidosis: A follow-up study. Lung.

[15] Drent M, Marcellis R, Lenssen A, De Vries J (2014). Association between physical functions and quality of life in sarcoidosis. Sarcoidosis Vasc. Diffuse Lung Dis..

[16] Jastrzębski D (2015). Fatigue in sarcoidosis and exercise tolerance, dyspnea, and quality of life. Adv. Exp. Med. Biol..

[17] Kouranos V, Jacob J, Wells AU (2015). Severe sarcoidosis. Clin. Chest Med..

[18] Strookappe B (2015). Benefits of physical training in sarcoidosis. Lung.

[19] Grongstad A, Vøllestad NK, Oldervoll LM, Spruit MA, Edvardsen A (2019). The effects of high- versus moderate-intensity exercise on fatigue in sarcoidosis. J. Clin. Med..

[20] Salazar A (2002). Corticosteroid therapy increases HDL-cholesterol concentrations in patients with active sarcoidosis and hypoalpha-lipoproteinemia. Clin. Chim. Acta.

[21] Telenga ED (2014). Untargeted lipidomic analysis in chronic obstructive pulmonary disease. Uncovering sphingolipids. Am. J. Respir. Crit. Care Med..

[22] Yan F (2017). Identification of the lipid biomarkers from plasma in idiopathic pulmonary fibrosis by lipidomics. BMC Pulm. Med..

[23] Landi C (2015). A functional proteomics approach to the comprehension of sarcoidosis. J. Proteomics.

[24] Toczylowska B, Jastrzebski D, Kostorz S, Zieminska E, Ziora D (2018). Serum lipidomics in diagnostics of sarcoidosis. Sarcoidosis Vasc. Diffuse Lung Dis..

[25] De Kleijn WP, De Vries J, Wijnen PA, Drent M (2011). Minimal (clinically) important differences for the Fatigue Assessment Scale in sarcoidosis. Respir. Med..

[26] Fletcher CM (1960). Standardized questionnaire on respiratory symptoms: A statement prepared and approved by the MRC Committee on the etiology of chronic bronchitis (MRC breathlessness score). BMJ.

[27] Miller M (2005). Standardization of spirometry. Eur. Respir. J..

[28] Graham BL (2017). ERS/ATS standards for single-breath carbon monoxide uptake in the lung. Eur. Respir. J..

[29] Przybyłowski T (2015). Polish Respiratory Society guidelines for the methodology and interpretation of the 6 minute walk test (6MWT). Pneumonol. Alergol. Pol..

[30] Singh SJ (2014). An official systematic review of the European Respiratory Society/American Thoracic Society: Measurement properties of field walking tests in chronic respiratory disease. Eur. Respir. J..

[31] Brown LE, Weir JP (2001). Asep procedures recommendation I: Accurate assessment of muscular strength and power. J. Exerc. Physiol..

[32] Gibson GJ (2002). ATS/ERS Statement on respiratory muscle testing. Am. J. Respir. Crit. Care Med..

[33] Spruit MA (2013). An official American Thoracic Society/European Respiratory Society statement: Key concepts and advances in pulmonary rehabilitation. Am. J. Respir. Crit. Care Med..

[34] Bligh EG, Dyer WJ (1959). A rapid method of total lipid extraction and purification. Can. J. Biochem. Physiol..

[35] Podlecka-Piętowska A (2019). Altered cerebrospinal fluid concentrations of hydrophobic and hydrophilic compounds in early stages of multiple sclerosis-metabolic profile analyses. J. Mol. Neurosci..

[36] Jonsson P (2015). Constrained randomization and multivariate effect projections improve information extraction and biomarker pattern discovery in metabolomics studies involving dependent samples. Metabolomics.

[37] Naughton DP (1993). A comparative evaluation of the metabolic profiles of normal and inflammatory knee-joint synovial fluids by high resolution proton NMR spectroscopy. FEBS Lett..

[38] Blundell CD, Reed MAC, Almond A (2006). Complete assignment of hyaluronan oligosaccharides up to hexasaccharides. Carbohydr. Res..

[39] Toczylowska B, Piotrowski M, Chalimoniuk M (2011). P-31 high resolution NMR spectroscopy in analysis of phosphate-containing compounds of bile. Biocybern. Biomed. Eng..

[40] Li J, Vosegaard T, Guo Z (2017). Applications of nuclear magnetic resonance in lipid analyses: An emerging powerful tool for lipidomics studies. Progr. Lipid Res..

[41] Bylesjo M (2006). OPLS discriminant analysis: Combining the strengths of PLS-DA and SIMCA classification. J Chemom..

[42] Ellis DI, Dunn WB, Griffin JL, Allwood JW, Goodacre R (2007). Metabolic fingerprinting as a diagnostic tool. Pharmacogenomics J..

[43] Weckwerth W, Morgenthal K (2005). Metabolomics: From pattern recognition to biological interpretation. Drug Discov. Today.

[44] Dobiasova M, Frohlich J (2001). The plasma parameter log (TG/HDL-C) as an atherogenic index: Correlation with lipoprotein particle size and esterification rate in apo B-lipoprotein-depleted plasma. (FERHDL). Clin. Biochem..

[45] Wheelock CE (2013). Application of ’omics technologies to biomarker discovery in inflammatory lung diseases. Eur. Respir. J..

[46] Salazar A, Pintó X, Mañá J (2001). Serum amyloid A and high-density lipoprotein cholesterol: Serum markers of inflammation in sarcoidosis and other systemic disorders. Eur. J. Clin. Investig..

[47] Vekic J (2013). Distribution of low-density lipoprotein and high-density lipoprotein subclasses in patients with sarcoidosis. Arch. Pathol. Lab. Med..

[48] Bargagli E (2011). Analysis of serum amyloid A in sarcoidosis patients. Respir. Med..

[49] Wang Y, Shen L, Xu D (2019). Aerobic exercise reduces triglycerides by targeting apolipoprotein C3 in patients with coronary heart disease. Clin. Cardiol..

[50] Fikenzer K, Fikenzer S, Laufs U, Werner C (2018). Effects of endurance training on serum lipids. Vascul. Pharmacol..

