# Supplementary material for: The effects of exercise training on lipid profile in patients with sarcoidosis
Source: Sci Rep. 2021 Mar 10;11:5551. doi: 10.1038/s41598-021-84815-4 (PMC7946908; doi:10.1038/s41598-021-84815-4)
Supplement: Supplementary file 1 — Supplementary Table S1. [file 41598_2021_84815_MOESM1_ESM.doc]

**Supplementary Table S1. Study group BMI and total cholesterol.**

| **Patient** | **Age** | **Gender** | **BMI** | **Total Cholesterol [mmol/L]** |
| --- | --- | --- | --- | --- |
| 1 | 59 | F | 24 | 7,78 |
| 2 | 35 | F | 25,6 | 6,5 |
| 3 | 60 | F | 28,7 | 6,9 |
| 4 | 62 | F | 36,5 | 7,8 |
| 5 | 45 | M | 31,6 | 7,24 |
| 6 | 59 | F | 27,3 | 6,66 |
| 7 | 38 | M | 31,3 | 7,9 |
| 8 | 37 | M | 30,1 | 7,21 |
| 9 | 43 | M | 23,4 | 6,5 |
| 10 | 37 | F | 22,6 | 6,2 |
| 11 | 52 | F | 31,1 | 7,3 |
| 12 | 41 | M | 18,9 | 6,15 |
| 13 | 41 | M | 26 | 7,18 |
| 14 | 35 | F | 23 | 6,23 |
